# Parasitic contamination of raw vegetables and fruits collected from selected local markets in Arba Minch town, Southern Ethiopia

**DOI:** 10.1186/s40249-016-0226-6

**Published:** 2017-03-07

**Authors:** Fitsum Bekele, Tamirat Tefera, Gelila Biresaw, Tsegaye Yohannes

**Affiliations:** 1 Department of Public Health, College of Health Sciences and Medicine, Wolaita Sodo University, P.O. Box 138, Wolaita Sodo, Ethiopia; 2 Department of Medical Laboratory Sciences and Pathology, College of Public Health and Medical Sciences, Jimma University, P.O. Box 378, Jimma, Ethiopia; 3 Department of Medical Laboratory Science, College of Health Sciences and Medicine Arba Minch University, P.O. Box 21, Arba Minch, Ethiopia

**Keywords:** Vegetable and fruits contamination, Intestinal parasite, Health education, Arba Minch, Ethiopia

## Abstract

**Background:**

One way that people get infected with intestinal parasites is through the consumption of contaminated vegetables and fruits. This study aimed at determining the prevalence and predictors of parasitic contamination of fruits and vegetables collected from four local markets in Arba Minch town, Southern Ethiopia.

**Methods:**

A cross-sectional study was conducted from 1 to 21 September 2014 to determine the level of parasitic contamination of fruits and vegetables sold in Arba Minch town. A total of 360 samples of different types of fruits and vegetables were soaked in physiological saline, followed by vigorous shaking with the aid of a mechanical shaker for 15 minutes and then examined using the sedimentation concentration technique.

**Results:**

Out of the 360 samples examined, 196 (54.4%) were contaminated with at least one type of parasite. *Ascaris lumbricoides* (20.83%) was the most frequently detected parasite and *Isospora belli* (3.06%) was the least frequently detected one*.* It was also observed that decreased parasitic contamination was significantly associated with washing the products before displaying it for selling (*P* < 0.001).

**Conclusions:**

The findings of this study provide evidence that there is a potentially high risk of acquiring parasitic infections from the consumption of raw vegetables and fruits in Arba Minch, Ethiopia. The authors believe that an effort should be made by the relevant bodies to reduce the rate of contamination of products with medically important parasites by educating the vendors and the community.

**Electronic supplementary material:**

The online version of this article (doi:10.1186/s40249-016-0226-6) contains supplementary material, which is available to authorized users.

## Multilingual abstracts

Please see Additional file 1 for translations of the abstract into six official working languages of the United Nations.

## Background

Intestinal parasitic infections are widely distributed throughout the world, endangering public health. Infections with medically important parasites (intestinal helminths and protozoa) are closely linked with conditions of poverty, unsafe water, crowded living conditions, lack of sanitation and hygiene [1, 2].

Food borne diseases continue to be a common and serious threat to public health all over the world and these diseases are a major cause of morbidity [3]. Outbreaks of human infections due to the consumption of raw fruits and vegetables have occurred with increased frequency during the past decade [3]. Studies have shown that *Ascaris lumbricoides*, *Cryptosporidium* spp., *Entamoeba histolytica, Enterobius vermicularis, Fasciola* spp., *Giardia intestinalis*, hookworm, *Hymenolepis* spp., *Taenia* spp.*, Trichuris trichiura*, and *Toxocara* spp., can infect humans who consume contaminated, uncooked, or improperly washed vegetables and fruits [4].

Many studies had been conducted to evaluate the role of raw vegetables in the transmission of intestinal parasites, for example, in Alexandria, Egypt; Tripoli, Libya; Riyadh, Saudi Arabia; Iraq; Tehran and Qazvin Province, Iran; and the Philippines [5–13]. All have stressed the importance of fruits and vegetables, particularly which are consumed raw and unwashed, in the transmission of medically important parasites.

Fruits and vegetables—by being a source of essential nutrients, vitamins, minerals, proteins, and fibers—play a major role in protecting the human body from a number of diseases. Consuming raw and improperly washed vegetables is a major way in which human pathogens are transmitted [10, 11]. Because of poor hygienic practices related to planting, harvesting, packing, transportation, and storage, fruits and vegetables can become easily contaminated with parasites [11].

In developing countries such as Ethiopia, poor sanitation, and substandard and crowded living conditions lead to an increased risk of acquiring parasitic infections. To the best of our knowledge, there is no published document about the level of parasitological contamination of fruits and vegetables in Arba Minch town, Southern Ethiopia. Therefore, this study was designed to determine the level of parasitic contamination of selected fruits and vegetables and associated factors in this location.

## Methods

### Study area

A cross-sectional study was conducted between 1 and 20 September, 2014 to determine the level of parasitic contamination of fruits and vegetables sold in selected local markets in Arba Minch town, Southern Ethiopia. Arba Minch is located 505 km south of the capital Addis Ababa at an altitude of 1 200–1 300 meters above sea level with an average annual temperature of 29.7 °C. The town experience two distinct wet seasons which occur from March to May followed by a lesser rainfall season in October to December. The average annual rainfall is 700 mm.

### Data collection

A pre-tested semi-structured questionnaire was used for collecting data on factors associated with parasitic contamination of fruits and vegetables such as: status of the produces [washed before display or not, freshly collected or stayed more than one day, source of water used for washing, educational status of the vendors]. Data on means of display and type of the market were recorded by simple observation. In each market, samples were collected under normal purchase conditions from randomly selected sellers. A total of 40 vendors participated in this study.

### Sample collection and analysis

Eight types of fruits and vegetables (*Persea americana (*Avocado*)*, *Lactuca serriola (*Lettuce*)*, *Brassica oleracea (*Cabbage*)*, *Daucus carota (*Carrot*)*, *Lycopersicon esculentum (*Tomato*)*, *Capsicum annuum(*Green pepper*)*, *Musa paradisiaca (*Banana*)*, and *Mangifera indica (*Mango*)*) were purchased from four conveniently located local markets, namely, Sikela (1 235 meters above sea level), Secha (1 300 meters above sea level), Yetnebersh (12 90 meters above sea level), and Konso-Sefer (1 275 meters above sea level) in Arba Minch town. An equal number of samples (45 each, total 360 samples) were collected from the markets.

Each sample was placed in a separate plastic bag and labeled with a unique number and its date of collection, and brought to the Arba Minch Hospital Leishmaniasis Research Centre Laboratory for parasitological analysis. Approximately 200 grams of each vegetable or fruit was soaked (for 15 minutes) in one liter of physiological saline, followed by vigorous shaking with the aid of a mechanical shaker [Vortex Genie 2] for 15 minutes. After overnight sedimentation in the washing solution, 15 milliliters of the sediment was transferred to a centrifuge tube using a sieve to remove undesirable matter. For concentrating the parasitic stages (ova, larvae, cysts, and oocysts), the tube was centrifuged at 3000 revolutions per minute for five minutes [13]. After centrifugation, the supernatant was decanted carefully without shaking. Then, the sediment was agitated gently by hand to redistribute the parasitic stages. Finally, the sediment was examined under a light microscope [Olympus CHT] using × 10 and × 40 objectives. Modified Ziehl-Neelsen stained smears were prepared to detect coccidian protozoan oocysts including *Cryptosporidium* spp., *Isospora belli*,and *Cyclospora cayetanensis* [14–16].

### Data analysis

Statistical analysis was performed with SPSS software version 16 (IBM, Chicago, IL, USA). Probability values were considered to be statistically significant when the calculated *P*-value was equal to or less than 0.05. The difference in parasitic contamination among the different categories was compared using the Pearson’s chi-square test (χ^2^) and Fisher’s exact test, where appropriate. Univariate and multivariate logistic regression analyses were performed to identify factors associated with parasitic contamination of the fruits and vegetables.

## Results

In this study, 196 samples were identified as being contaminated with at least one type of parasite; the overall contamination rate was 54.4%. Table 1 summarizes what percentage of each sample group was infected: 48.9% of the *Capsicum annuum* was infected, 66.7% of *Brassica oleracea*, 37.8% of *Persea americana,* 60.0% of *Lactuca serriola*, 62.2% of *Daucus carota*, 71.1% of *Lycopersicon esculentum*, 48.9% of *Musa paradisiacal*, and 40.0% of *Mangifera indica*.

**Table 1:** Frequency distribution of parasitological contamination of fruits and vegetables inselected markets inArba Minch town 1 to 22 September 2014

Type of produce	Number examined	Number positive [%]	Number of parasite species detected			
			One [%]	Two [%]	Three [%]	Four [%]
*Capsicum annuum*	45	22 [48.9]	16 [35.6]	4 [8.9]	2 [4.4]	0
*Mangiferaindica*	45	18 [40.0]	14 [31.1]	3 [6.7]	1 [2.2]	0
*Perseaamericana*	45	17 [37.8]	14 [31.1]	1 [2.2]	1 [2.2]	1 [2.2]
*Lycopersiconesculentum*	45	32 [71.1]	27 [60.0]	3 [6.7]	1 [2.2]	1 [2.2]
*Daucuscarota*	45	28 [62.2]	23 [51.1]	3 [6.7]	2 [4.4]	0
*Lactucaserriola*	45	27 [60.0]	25 [55.6]	2 [4.4]	0	0
*Brassica oleracea*	45	30 [66.7]	26 [57.8]	3 [6.7]	1 [2.2]	0
*Musa paradisiaca*	45	22 [48.9]	19 [42.2]	2 [4.4]	1 [2.2]	0
Total	360	196 [54.4]	164 [45.6]	21 [46.7]	9 [20.0]	2 [4.4]

The parasites detected included ova of *A. lumbricoides*, *Toxocara* spp., *Hymenolepis nana*, and *H. diminuta*; oocysts of *Cyclospora, I. belli*, and *Cryptosporidium* spp.; and cysts of *G. intestinalis* and *E. histolytica/dispar*. Table 2 shows that *A. lumbricoides* (20.83%) was the most frequently detected parasite, followed by *Toxocara* (15.83%), *Hymenolepis nana* (15.56%), *E. histolytica/dispar* (14.44%), *G. intestinalis* (10.0%), *H. diminuta* (7.78%), *Cyclospora* (6.94%), *Cryptosporidium* (4.72%), and *I. belli* (3.06%).

**Table 2:** Prevalence of intestinal parasites in fruits and vegetables sold at four local markets in Arba Minch town from 1 to 22 September 2014

Detected parasite	Frequency	Prevalence
*Ascaris lumbricoides*	75	20.83%
*Toxocara* spp.	57	15.83%
*Hymenolepis nana*	56	15.56%
*Entamoeba histolytica/dispar*	52	14.44%
*Giardia intestinalis*	36	10.00%
*H. diminuta*	28	7.78%
*Cyclospora* spp.	25	6.94%
*Cryptosporidium*	17	4.72%
*Isospora belli*	11	3.06%

Contamination with more than one parasite species was observed in the fruit and vegetable samples examined in this study. Almost half (46.7%) of the total samples were contaminated with two species of parasites, while 20% of the samples were contaminated with three species of parasites and quadruple parasitic contamination was observed in 4.4% of the samples. The parasitic contamination rate of the different fruits and vegetables was significantly different (*P* = 0.03) (see Table 3).

**Table 3:** Factors associated with parasitic contamination of fruits and vegetables sold in selected markets of Arba Minch town from 1 to 22 September 2014 determined using the chi-square test

Variable	Result of parasitological analysis			
	Positive [%]	Total	χ^2^ value	*P*-value
*Educational status of vendors*				
No formal education	115 [63.9]	180		
Primary education	70 [46.7]	150	14.07	0.001
Secondary education	11 [36.7]	30		
Total	196 [54.4]	360		
*Market*				
Secha	50 [55.6]	90		
Sikela	48 [53.3]	90		
Konso-sefer	60 [66.7]	90	4.98	0.064
Yetnebersh	38 [42.2]	90		
Total	196 [54.4]	360		
*Type of produce*				
*Lactucaserriola*	27 [60]	45		
*Mangiferaindica*	18 [40]	45		
*Perseaamericana*	17 [37.8]	45		
*Lycopersiconesculentum*	32 [71.2]	45	8.79	0.03
*Daucuscarota*	28 [62.3]	45		
*Capsicum annuum*	22 [48.9]	45		
*Musa paradisiaca*	22 [48.9]	45		
*Brassica oleracea*	30 [66.7]	45		
Total	196 [54.4]	360		
*Means of display*				
On the floor	111 [55.5]	200		
On tops of tables	29 [39.7]	73	5.66	0.722
On wheelbarrow	36 [41.4]	87		
Total	196 [54.4]	360		
*Washed before display*				
Yes	40 [38.5]	104		
No	156 [60.9]	256	6.78	0.001
Total	196 [54.4]	360		
*Source of water for washing*				
Pipe	21 [35.0]	60		
River	14 [48.3]	29	1.771	0.441
Well	2 [28.6]	7		
Total	37 [38.5]	96		
*Market type*				
Grocery	36 [60]	60		
Open market	160 [53.3]	300	0.39	0.88
Total	196 [54.4]	360		

Further analysis with binary logistic regression revealed that, compared to *Persea americana*, *Lycopersicon esculentum* was significantly contaminated (adjusted odds radio, A*OR* = 3.4, 95% confidence interval, *CI* [1.6, 9.9]) (see Table 4).

**Table 4:** Binary logistic regression of factors associated with parasitic contamination of fruits and vegetables sold in selected markets of Arba Minch town from 1 to 22 September 2014

Variables	Pos. (%)	Laboratory result for parasitic contamination C*OR* (95% *CI*) A*OR* (95% *CI*)	
*Sample type*			
*Mangiferaindica*	18 [40]	1.0 (0.3, 2.4)	1.0 (0.2, 2.6)
*Brassica oleracea*	30 [66.7]	2.4 (1.2, 5.8)*	2.2 (1.0, 5.6)
*Daucuscarota*	28 [62.3]	1.7 (0.7, 4.4)	1.7 (0.7, 4.6)
*Capsicum annuum*	22 [48.9]	1.3 (0.6, 2.6)	1.3 (0.5, 2.8)
*Lactucaserriola*	27 [60]	1.8 (0.8, 4.3)	1.8 (0.7, 4.4)
*Lycopersiconesculentum*	32 [71.2]	2.5 (1.1, 5.9)*	3.4 (1.6, 9.9)
*Musa paradisiaca*	22 [48.9]	1.2 (0.5, 2.7)	1.2 (0.4, 2.9)
Perseaamericana**	17 [37.8]		
*Washed before display*			
No	156 [38.5]	3.1 (1.8, 4.4)*	3.6 (1.9, 4.6)
Yes**	40 [60.9]		
*Market*			
Secha	50 [55.6]	0.5 (0.3, 1.0)	0.5 (0.3, 0.9)
Sikela	48 [53.3]	0.6 (0.4, 1.0)	0.5 (0.3, 1.1)
Konso-Sefer	60 [66.7]	1.1 (0.6, 2.0)	1.1 (0.5, 2.1)
Yetnebersh**	38 [42.2]		
*Means of display*			
On the floor	111 [55.5]	1.2 (0.5, 5.1)	1.7 (0.4, 5.1)
On wheelbarrow	36 [41.4]	1.5 (0.3, 4.9)	1.6(0.4, 5.5)
On table**	29 [39.7]		
*Market type*			
Grocery	36 [60]	1.2 (0.6, 1.9)	1.2 (0.6, 2.1)
Open market	160 [53.3]**		
*Vendor’s educational status*			
No formal education	115 [63.9]	1.2 (0.6, 2.6)	1.3 (0.6, 2.8)
Primary education	70 [46.7]	0.9 (0.4, 1.8)	0.9 (0.6, 1.9)
Secondary education**	11 [36.7]		

*Significant at *P*-value of 0.05; **reference category

The results also showed that samples collected from Konso-Sefer (66.7%) had the highest contamination rate, followed by samples collected from Secha (55.6%), Sikela (53.3%), and Yetnebersh (42.2%). However, the contamination rate of samples collected from the different markets was statistically insignificant (*P* = 0.064) (see Table 3). Samples were collected from both open markets and groceries. Samples from groceries comprised 18.4% of the positive samples, while 81.6% of the contaminated products were from open markets; the difference was not statistically significant (*P* = 0.88) (see Table 3).

This study also assessed which factors were associated with contamination of fruits and vegetables by conducting interviews with the vendors in the markets. Vendors were asked about their educational status and it was revealed that the majority (50%) of the vendors had no formal education, while 42% had primary education and only 8% had secondary education. There was significant association between the education level of vendors and the parasitic contamination rate of the produce they were selling (*P* = 0.001) (see Table 3).

Among the factors associated with parasitic contamination of fruits and vegetables is the act of washing products before displaying it for sale. This study showed that the majority (79.6%) of the products were not washed before display, with only 20.4% being washed. The cross tabulation of washing the products before display for sale and the results of the parasitological analysis showed a significant difference in the contamination rate between washed and unwashed products (*P* = 0.001) (see Table 3). Compared to washed products, the odds of unwashed products becoming contaminated with at least one parasite was 3.6 times higher (A*OR* = 3.6, 95% *CI* [1.9, 4.6]) (see Table 4).

The means of display was also assessed for association with parasitic contamination of fruits and vegetables; a statistically insignificant association was observed (*P* = 0.072).

The sources of water used for washing products were also examined: pipe water (62.5%), well water (30.2%), and river water (7.3%). The analysis revealed that 35%, 48.3%, and 28.6% of the produce washed by pipe water, well water, and river water was contaminated with at least one parasite species, respectively. There was no significant difference observed in the contamination rate between the products washed by water from different sources (*P* = 0.441) (see Table 3).

## Discussion

Like many tropical countries, intestinal parasites are widely distributed in Ethiopia because of the favorable climate and unsanitary conditions that facilitate fecal pollution of water, foodstuffs, and soil [2, 11]. The market chain lends itself to fruits and vegetables passing through several hands and as a result they might become contaminated with enteric bacteria, viruses, and parasitic pathogens [16].

The present study attempted to assess the level of contamination and prevalence of different intestinal parasites in various fruits and vegetables sold in local markets of Arba Minch, Southern Ethiopia. The overall parasitic contamination rate was found to be 54.4%, which is in agreement with other findings from southwest Ethiopia and elsewhere [11–15]. It is also, however, higher than that reported in similar studies from, Egypt, Ethiopia and Gaza governorates [5, 17–20]. The incongruity between this study and others might be attributed to the variations in geographical locations, climatic and environmental conditions, differences in the sample size, the techniques used, poor post-harvest handling method and socioeconomic status.

*Lycopersicon esculentum* (71.2%) was found to be the most frequently contaminated product, followed by *Brassica oleracea* (66.7%), *Daucus carota* (62.3%), *Lactuca serriola* (60.0%), *Capsicum annuum* and *Musa paradisiaca* (48.9% each).*Mangifera indica* (40.0%) and *Persea americana* (37.8%) were found to be the least contaminated. The variation in contamination between the products might be explained by the fact that vegetables such as cabbage, carrot, and lettuce have larger and uneven surfaces, which make the parasites attach more easily to the surface. The smooth surface of *Capsicum annuum*, *Persea americana*, and *Mangifera indica* might reduce the rate of parasitic attachment hence explaining the lower contamination rate observed in this study [5, 21].

In this study, *A. lumbricoides* was the most frequently detected parasite with a prevalence of 20.83%. The predominance of *A. lumbricoides* agrees with studies conducted in Philippines and Kenya [11, 21]. This dominance might be associated with this parasite’s ubiquitous distribution, the high number of eggs produced by the fecund female parasite which contributes to the parasite ubiquitous distribution, and the strong and resistant nature of the eggs that enables them to survive unfavorable conditions. The eggs can survive in the absence of oxygen, live for two years at 5–10 °C, and be unaffected by desiccation for two to three weeks [22].

The second most prevalent contaminant was *Toxocara* species with the prevalence of 15.83%. This dominance might be attributed to the fertility of *Toxocara* female adults producing up to 10 000 eggs daily and the nature of the eggs, which may survive for up to ten years resisting harsh environmental conditions [23, 24].

*H. nana* was the third most frequently detected parasite in this study, with a prevalence of 15.56%. This finding is higher than the finding of 8.3% reported in Jimma, Ethiopia and also a study done in Banha, Egypt, which reported a prevalence of 2.8%. The difference observed might be due to difference in climatic conditions and geographical location [15, 25].

In this study, no ova of hookworm are detected. This is in agreement with other studies conducted in Jimma, Ethiopia and Banha, Egypt [15, 25]. It is known that hookworm has a very short life span in the soil and this characteristic might have contributed to its absence in the present study [26, 27]. On the contrary, a study done in Jos, Nigeria has reported the contamination of vegetables with *hook worm* species [28]. The differences might be attributed to differences in geographical locations, climatic conditions, and types of soil [28, 29].

Various studies support the findings of this study in regards to contamination of fruits and vegetables with *G. intestinalis*. These include a study done in Tripoli, Libya, and Ardabil, Iran, reporting a prevalence of 10% and 7%, respectively [7, 30]. Our findings on *G. intestinalis* are also in agreement with a recent study done in Ahar, Iran, which reported a prevalence of 10% [31].

In general, comparing with other similar studies done in the region, a higher rate of parasitic contamination of fruits and vegetables was observed in this study and this could be attributed to many factors such as geographical location, type and number of samples examined, methods used for detection of the intestinal parasites, type of water used for irrigation, and post-harvesting handling methods, which are different from one country to another. In addition to the above factors, individual hygienic habits, sanitary facilitations, and climatic conditions all play a significant role [3].

Contamination with multiple species was observed in all kinds of fruits and vegetables in this study. This might indicate the possibility of a high-level contamination of fruits and vegetables, which perhaps results in multiple parasitic infections in humans. It might also indicate the persistence of intestinal parasitic infections in the area [13].

Even though it was statistically insignificant, the contamination rate was different for the samples collected from different markets. Samples collected from Konso-Sefer showed a higher rate of contamination. This might be associated with the way the products are displayed and the act of washing produce before display. The majority (55.5%) of the samples was displayed on the floor, which exposes them to flies, and 60.9% of the samples were not washed before display. It has been reported that flies can act as a vector for parasites such as *Cryptosporidium parvum* [32].

The odds of unwashed produce before display becoming contaminated with at least one parasite was 3.6 times higher (A*OR* = 3.6, 95% *CI*[1.9, 4.6]) when compared with those washed before display. This might be due to the risk of contamination of the produce during transportation and other post-harvest related activities [15, 32].

## Conclusion

The high prevalence of intestinal parasites in the fruits and vegetables consumed in Arba Minch indicates that produce is one of the sources leading to parasitic infections among the public. The authors believe that prevention of contamination remains the most effective way of reducing fruit- and vegetable-borne parasitic infections, and this can be achieved by proper washing of vegetables, improved hygienic practices of vegetable handlers, and improvements in sanitation standards. Comprehensive health education should also be given to vendors and farmers. Vendors should ensure that produce does not make contact with the soil when displaying for selling. In addition, other research must be done to evaluate the level of parasitic contamination of farm products, water, and soil in which fruits and vegetables are cultivated.

## Additional file

Additional file 1: Multilingual abstracts in the six official working languages of the United Nations. (PDF 769 kb)

## Abbreviations

AOR: Adjusted odds ratio
CI: Confidence interval
COR: Crude odds ratio

## References

[1] Okyay P, Ertug S, Gultekin B, Onen O, Beser E (2004). Intestinal parasites prevalence and related factors in school children, a western city sample-Turkey. BMC Public Health..

[2] Wegayehu T, Tsalla T, Seifu B, Teklu T (2013). Prevalence of intestinal parasitic infections among highland and lowland dwellers in Gamo area, South Ethiopia. BMC Public Health..

[3] de W Blackburn C, McClure PJ (2002). Foodborne pathogens: hazards, risk analysis and control.

[4] Kozan E, Gonenc B, Sarimehmetoglu O, Aycicek H (2005). Prevalence of helminth eggs on raw vegetables used for salads. Food Control..

[5] Said DE (2012). Detection of parasites in commonly consumed raw vegetables. Alexandria J Med..

[6] Hassan A, Farouk H, Abdul-Ghani R (2012). Parasitological contamination of freshly eaten vegetables collected from local markets in Alexandria, Egypt: A preliminary study: a preliminary study. Food Control.

[7] AbougrainAK NMH, Madi NS, Saied MM, Ghenghesh KS (2010). Parasitological contamination in salad vegetables in Tripoli-Libya. Food Control.

[8] Al-Megrin WAI (2010). Prevalence of intestinal parasites in leafy vegetables in Riyadh, Saudi Arabia. Int J Zool Res..

[9] Hadi AM (2011). Isolation and identification of intestinal parasites from vegetables from different markets of Iraq. Bull Iraq Natural History Museum.

[10] Gharavi MJ, Jahani MR, Rokni MB (2002). Parasitic contamination of vegetables from farms and markets in Tehran. Iran J Public Health.

[11] Sia Su GL, Mariano CMR, Matti NSA, Ramos GB (2012). Assessing parasitic infestation of vegetables in selected markets in Metro Manila, Philippines. Asian Pacific J Trop Dis..

[12] Shahnazi M, Jafari-Sabet M (2010). Prevalence of parasitic contamination of raw vegetables in villages of Qazvin Province, Iran. Foodborne Pathog Dis..

[13] Omowaye OS, Audu PA (2012). Parasites contamination and distribution on fruits and vegetables in Kogi, Nigeria. CIBTech J Bio-Protocols.

[14] Idahosa OT (2011). Parasitic Contamination of Fresh Vegetables Sold in Jos Markets. Global J Med Res.

[15] Tefera T, Biruksew A, Mekonnen Z, Eshetu T (2014). Parasitic contamination of fruits and vegetables collected from selected local markets of Jimma town, southwest Ethiopi. Int Sch Res Notices..

[16] Cheesbrough M (2009). District Laboratory Practice in Tropical Countries, part 1.

[17] TomassZ KD (2012). Parasitological contamination of wastewater irrigated and raw manure fertilized vegetables in Mekelle city and its suburb, Tigray, Ethiopia. Momona Ethiopian J Sci..

[18] Al-Shawa RM, Mwafy SN (2007). The enteroparasitic contamination of commercial vegetables in Gaza Governorates. J Infect Dev Countries.

[19] Ishaku A, Ishakeku D, Agwale S (2013). Prevalence of parasitic contamination of some edible vegetables sold at alhamis market in lafiametropolist. Scholar J Biotechnol.

[20] Ogbolu DO, Alli OA, Ogunleye VF, Olusoga OF, Olaosun I (2009). The presence of intestinal parasites in selected vegetables from open markets in south western Nigeria. Afr J Med Med Sci.

[21] Nyarango RM, Aloo PA, Kabiru EW, Nyanchongi BO (2008). The risk of pathogenic intestinal parasite infections in Kisii Municipality, Kenya. BMC Public Health..

[22] Roberts AD, Satoskar AR, Simon GL, Hotez PJ, Tsuji M (2009). Ascariasis: Introduction and Epidemiology and Transmission. Medical Parasitology.

[23] Sunil B, Thomas DR, Latha C, Shameem H (2014). Assessment of parasitic contamination of raw vegetables in Mannuthy, Kerala state, India. Vet World.

[24] Klapec T, Borecka A (2012). Contamination of vegetables, fruits and soil with geohelmints eggs on organic farms in Poland. Ann Agric Environ Med.

[25] Ahmad Eraky MA, Rashed S ,Nasr M, Azza ,El-Hamshary M, El-Ghannam AS. Parasitic contamination of commonly consumed fresh leafy vegetables in Benha, Egypt. J Parasitol Res. 2014; 2014: 613960. doi: 10.1155/2014/613960 10.1155/2014/613960 PMC4084512 25024845

[26] Uga S, Hoa NT, Noda S, Moji K, Cong L, Aoki Y (2009). Parasite egg contamination of vegetables from a suburban market in Hanoi, Vietnam. Nepal Med Coll J..

[27] Augustine DL (1922). Investigations on the control of hookworm disease. X. Experiments on the length of life of infective hookworm larvae in soil. Am J Hygiene.

[28] Damen JG, Banwat EB, Egah DZ, Allanana JA (2007). Parasitic contamination of vegetables in Jos, Nigeria. Ann Afr Med..

[29] Mabaso MLH, Appleton CC, Hughes JC, Gouws E (2003). The effect of soil type and climate on hookworm (Necator americanus) distribution in KwaZulu‐Natal, South Africa. Trop Med Int Health..

[30] Daryani A, Ettehad GH, Sharif M, Ghorbani L, Ziaei H (2008). Prevalence of intestinal parasites in vegetables consumed in Ardabil, Iran. Food Control..

[31] Joghataei A, Balarak D, Mahdavi Y, Modrek JM (2016). Prevalence of parasitic contamination of raw vegetables in Ahar, Iran. Int J Analyt Pharmaceut Biomed Sci.

[32] Meerburg BG, Vermeer HM, Kijlstra A (2007). Controlling risks of pathogen transmission by flies on organic pig farms: a review. Outlook Agriculture.

