# Supplementary material for: Parasitic contamination of raw vegetables and fruits collected from selected local markets in Arba Minch town, Southern Ethiopia
Source: Infect Dis Poverty. 2017 Mar 7;6:19. doi: 10.1186/s40249-016-0226-6 (PMC5340032; doi:10.1186/s40249-016-0226-6)
Supplement: Additional file 1: — Multilingual abstracts in the six official working languages of the United Nations. (PDF 769 kb) [file 40249_2016_226_MOESM1_ESM.pdf]

Translation of the abstract into the six official working languages of the United Nations

التلوث الطفيلي للخضروات النيئة والفواكة المجمعة من أسواق محلية مختارة في بلدة أربا منش، جنوب إثيوبيا

Fitsum Bekele, Tamirat Tefera, Gelila Biresaw, Tsegaye Yohannes

ملخص
خلفية: من أحد الطرق التي يصاب فيها الأشخاص بالطفيليات المعوية هي من خلال استهلاك الخضروات والفواكه الملوثة. تهدف هذه الدراسة إلى تحديد مدى انتشار التلوث الطفيلي في الفواكه والخضروات المجمعة من أربع أسواق محلية في بلدة أربا منش، جنوب إثيوبيا والتنبوء بها.
الأساليب: أجريت دراسة شاملة لعدة قطاعات من فترة 1 سبتمبر إلى 21 سبتمبر 2014 لتحديد مستوى التلوث الطفيلي للفواكة والخضروات المباعة في بلدة أربا منش. نُقعت 360 عينة من أنواع مختلفة من الفواكة والخضروات في محلول فسيولوجي ثم تعرضت لهز قوي بواسطة هزاز آلي لمدة 15 دقيقية ثم فُحصت باستخدام تقنية تركيز الترسيب.
النتائج: من بين 360 عينة تم فحصها، 196 من العينات (54.4%) كانت ملوثة بنوع واحد من الطفيليات على الأقل. كان *الصفر الخراطيني* (20.83%) أكثر الأنواع الطفيليات شيوعًا التي كُشف عنها وأقلها شيوعًا*كان داء متماثلات البوائغ* بنسبة (3.06%). كما لوحظ بانخفاض التلوث الطفيلي كثيرًا عند غسل المنتج قبل عرضه للبيع (التلوث<0.001).
الخلاصة: تقدم نتائج هذه الدراسة دليل بأن هناك مخاطر عالية محتملة من اكتساب العدوى الطفيلية من خلال استهلاك الخضروات النيئة والفواكة في بلدة أربا مينش، جنوب إثيوبيا. يؤمن المؤلفون أنه يجب على الجهات المعنية بذل الجهد للحد من نسبة تلوث المنتج من خلال الطفيليات المهمة طبيًا وذلك عن طريق تثقيف البائعين والمجتمع.

Translated from English version into Arabic by Yosra Montasser, through

TRANSLATORS
WITHOUT BORDERS

埃塞俄比亚南部阿尔巴门奇镇当地市场生鲜蔬菜和水果的寄生虫污染情况

Fitsum Bekele, Tamirat Tefera, Gelila Biresaw, Tsegaye Yohannes

摘要
引言：人体感染肠道寄生虫的途径之一是食用污染的蔬菜和水果。本研究旨在确定从埃塞俄比亚南部阿尔巴门奇镇的 4 个当地市场收集的水果和蔬菜中寄生虫感染情况和预测因素。
方法：2014 年 9 月 1 日至 21 日，通过横断面研究以确定在阿尔巴门奇镇出售的水果和蔬菜的寄生虫感染程度。将 360 份不同类型的水果和蔬菜样品浸泡在生理盐水中，并借助机械振荡器剧烈摇动 15 min，然后利用沉降浓缩技术进行检查。
结果：在 360 份受测样品中，196 份（54.4%）至少被一种寄生虫污染。 蛔虫（*Ascaris lumbricoides*，20.83%）是检出率最高的寄生虫，贝氏等孢球虫（*Isospora belli*，3.06%）的检出率最低。寄生虫污染减少与产品销售前洗涤有显著相关性（*P*<0.001）。
结论：本研究结果表明，在埃塞俄比亚南部阿尔巴门奇镇，食用生鲜蔬菜和水果引发寄生虫感染的潜在风险很高。作者认为，相关机构应努力通过教育供应商和社区居民来降低农贸产品的主要医学寄生虫感染率。

Translated from English version into Chinese by Jin Chen, edited by Pin Yang

CDC CHINESE CENTER FOR DISEASE CONTROL AND PREVENTION
NATIONAL INSTITUTE OF PARASITIC DISEASES

Contamination parasitaire des fruits et légumes crus collectés sur une sélection de marchés locaux à Arba Minch, dans le sud de l'Éthiopie

Fitsum Bekele, Tamirat Tefera, Gelila Biresaw, Tsegaye Yohannes

Résumé

Contexte: La consommation de fruits et légumes contaminés est l'une des causes d'infection par les parasites intestinaux. Cette étude vise à déterminer la prévalence et les indicateurs de contamination parasitaire des fruits et légumes collectés sur quatre marchés locaux à Arba Minch, dans le sud de l'Éthiopie.
Méthodes: Une étude transversale a été menée du 1er au 21 septembre 2014 pour déterminer le niveau de contamination parasitaire des fruits et légumes vendus à Arba Minch. Ce sont au total 360 échantillons de différents types de fruits et légumes qui ont été plongés dans du sérum physiologique puis vigoureusement agités à l'aide d'un agitateur mécanique pendant 15 minutes, et enfin examinés selon la technique de concentration de la sédimentation.
Résultats: Sur les 360 échantillons examinés, 196 (soit 54,4%) étaient contaminés par au moins un type de parasite. L'*Ascaris lumbricoides* (20,83%) était le parasite le plus fréquemment détecté et l'*Isospora belli* (3,06%) le moins fréquemment. On a également constaté que la contamination parasitaire baissait de manière significative lorsque la denrée était lavée avant d'être mise en vente ($P<0,001$).
Conclusion: Les résultats de cette étude font apparaître un risque potentiellement élevé d'infections parasitaires lié à la consommation de fruits et légumes crus à Arba Minch, en Éthiopie. Les auteurs estiment que les organismes concernés devraient s'efforcer de réduire le taux de contamination des denrées porteuses de parasites considérés comme importants d'un point de vue médical en sensibilisant les vendeurs et la communauté.

Translated from English version into French by celote75, through

TRANSLATORS
WITHOUT BORDERS

Паразитарное загрязнение сырых овощей и фруктов на рынках города Арба-Мынча на юге Эфиопии

Fitsum Bekele, Tamirat Tefera, Gelila Biresaw, Tsegaye Yohannes

Аннотация
Базовая проблематика. Одним из путей заражения кишечными паразитами является употребление загрязненных овощей и фруктов. Целью данного исследования является определение распространенности и прогностических показателей паразитарного загрязнения фруктов и овощей, взятых с рынков города Арба-Мынча на юге Эфиопии.
Методы. Для определения уровня паразитарного загрязнения фруктов и овощей, продаваемых в городе Арба-Мынче, с 1 по 21 сентября 2014 года проводилось перекрестное исследование. Всего 360 образцов различных видов фруктов и овощей поместили в физиологический раствор, который в течение 15 минут энергично встряхивали с помощью механической мешалки, а затем исследовали седиментационным методом.
Результаты. Из 360 исследованных образцов 196 (54,4%) были заражены минимум одним видом паразитов. Наиболее часто обнаруживаемым паразитом стал вид *Ascaris lumbricoides* (20,83%), а *Isospora belli* (3,06%) — наименее часто обнаруживаемым. Было также отмечено, что снижение уровня паразитарного загрязнения в значительной степени связано с промывкой продуктов перед выставлением на прилавки ($P<0,001$).
Выводы. Результаты данного исследования свидетельствуют о том, что существует потенциально высокий риск заражения паразитарными инфекциями при употреблении сырых овощей и фруктов в Арба-Мынче в Эфиопии. Авторы исследования считают, что соответствующим органам необходимо предпринимать меры по снижению уровня заражения продуктов клинически значимыми видами паразитов путем просвещения продавцов и сообществ.

Translated from English version into Russian by Anna Romanenko, through

TRANSLATORS
WITHOUT BORDERS

Contaminación parasitaria de verduras y frutas crudas procedentes de diversos mercados de la ciudad de Arba Minch (Etiopía meridional)

Fitsum Bekele, Tamirat Tefera, Gelila Biresaw, Tsegaye Yohannes

Resumen

Antecedentes: el consumo de frutas y verduras contaminadas representa una forma de infección parasitaria intestinal. El objetivo de este estudio consiste en determinar la prevalencia y los factores predisponentes de la contaminación parasitaria de frutas y verduras procedentes de cuatro mercados de la ciudad de Arba Minch (Etiopía meridional).

Métodos: se realizó un estudio transversal desde el 1 hasta el 21 de septiembre de 2014 para determinar el nivel de contaminación parasitaria de la fruta y verdura vendidas en la ciudad de Arba Minch. Se sumergió un total de 260 muestras de diferentes tipos de fruta y verdura en suero fisiológico, tras lo cual se agitaron vigorosamente con un agitador mecánico durante 15 minutos y se examinaron mediante el método de concentración por sedimentación.

Resultados: de las 360 muestras examinadas, 196 (54,4 %) estaban contaminadas con al menos un tipo de parásito. *Ascaris lumbricoides* (20,83 %) fue el detectado con mayor frecuencia, e *Isospora belli* (3,06 %), el menos común. Se observó asimismo que la disminución de la contaminación parasitaria se asocia de forma significativa con que el producto haya sido lavado antes de su puesta en venta ($P < 0{,}001$).

Conclusión: lo descubierto en este estudio evidencia la existencia de un riesgo potencialmente alto de contraer una infección parasitaria por el consumo de frutas y verduras crudas en Arba Minch (Etiopía). Los autores creen que los órganos competentes deben hacer un esfuerzo por reducir las tasas de contaminación de los productos agrícolas con parásitos con relevancia clínica mediante la educación de los vendedores y de la comunidad.

Translated from English version into Spanish by Gloria Estevan Alcaide, through

TRANSLATORS
WITHOUT BORDERS
